# Acute tuft cell ablation in mice induces malabsorption and alterations in secretory and immune cell lineages in the small intestine

**DOI:** 10.14814/phy2.70264

**Published:** 2025-03-06

**Authors:** Michael Momoh, Francisca Adeniran, Cynthia Ramos, Kathleen E. DelGiorno, Hiroshi Seno, Joseph T. Roland, Izumi Kaji

**Affiliations:** ^1^ Section of Surgical Sciences and Epithelial Biology Center Vanderbilt University Medical Center Nashville Tennessee USA; ^2^ Department of Cell and Developmental Biology Vanderbilt University Nashville Tennessee USA; ^3^ Department of Gastroenterology and Hepatology Kyoto University Graduate School of Medicine Kyoto Japan

**Keywords:** epithelial cell differentiation, glucose absorption, mouse model, tuft cell

## Abstract

Intestinal tuft cells have recently been the focus of many studies due to their function in chemosensation and type 2 immunity in human gastrointestinal diseases. This study investigated the impact of acute tuft cell loss on intestinal physiological function. Tuft cell deletion was induced in *DCLK1‐IRES‐GFP‐CreERT2/+*;*Rosa‐DTA* (DCLK1‐DTA) mice by a single tamoxifen injection, concomitant with littermate controls. Transient deletion of intestinal and biliary tuft cells was maximal on day 4 and recovered by day 7 post tamoxifen. DCLK1‐DTA mice presented with significantly shortened small intestinal length and greater body weight loss by day 4. The activity of Na^+^‐dependent glucose transporter 1 (SGLT1) and cystic fibrosis transmembrane regulator (CFTR) was reduced. Correlated with tuft cell reduction, the frequency of cholecystokinin (CCK)^+^ enteroendocrine and intermediate secretory cells, which co‐express Paneth and goblet cell markers, was increased. In the lamina propria, fewer mast cells and leukocytes were found in the Day 4 DCLK1‐DTA mice compared to controls. Ablation of tuft cells may induce nutrient malabsorption through alterations in epithelial cell proliferation and differentiation, along with changes in the mucosal defense response. These observations identify a new role for tuft cells in regulating intestinal absorption and mucosal regeneration.

## 1 INTRODUCTION

Tuft cells are rare chemosensory cells found in many organ systems, including the gastrointestinal (GI) tract. Known for their distinct bottle‐shaped appearance with a ‘tuft‐like’ bundle of microvilli on the apical surface, they are defined by the expression of the chemosensory cell‐specific transcription factor POU domain, class 2, transcription factor 3 (POU2F3) (Huang et al., 2018; Matsumoto et al., 2011). In mouse tissues, tuft cells are marked by the neuronal protein doublecortin‐like kinase 1 (DCLK1) (Gerbe et al., 2011). Although this marker is shared with other cell types, DCLK1 expression in the epithelium has become an established marker for tuft cells in mice (McKinley et al., 2017; Yi et al., 2019). Tuft cells in digestive organs also express choline acetyltransferase (ChAT), which is an essential enzyme for synthesizing the neurotransmitter acetylcholine (ACh) (Schutz et al., 2019). Non‐neuronal ACh release from tuft cells is an important secretagogue for activating epithelial defense responses (Billipp et al., 2024; Ndjim et al., 2024). Intestinal tuft cells possess similar characteristics to taste cells, utilizing key components involved in the taste signaling cascade, including transient receptor potential channel M5 (TRPM5), alpha‐gustducin, and taste receptor type 1 members (TAS1Rs) (Bezencon et al., 2008). Whole‐body knockout of alpha‐gustducin or TAS1R3 in mice indicates that these sugar sensors regulate glucose transporter expression in the small intestine (Moran et al., 2021). However, the involvement of tuft cells in this regulatory mechanism has not been clarified.

In the intestinal mucosa, tuft cells are implicated in a sensory role in initiating a type 2 immune response upon challenge by parasitic helminth infection or microbiota dysregulation (Gerbe et al., 2016; von Moltke et al., 2016). Genetically modified mice lacking chemosensory pathway components (TRPM5 or alpha‐gustducin deficient strains) fail to activate a type 2 immune response upon parasite colonization (Howitt et al., 2016). Activated tuft cells secrete the cytokine interleukin 25 (IL‐25), which stimulates tissue resident group 2 innate lymphoid cells (ILC2s) in the lamina propria to produce IL‐13. IL‐13 stimulates the proliferation of the stem cells in the crypts, inducing the expansion of tuft and goblet cell populations (Howitt et al., 2016; von Moltke et al., 2016). This feed‐forward circuit drives parasite clearance in the intestinal mucosa through increased tuft cell‐derived ACh and goblet cell mucin secretion. The absence of sensory tuft cells leads to higher parasite burden in mice, significantly slowing down this “weep and sweep” process (Lei et al., 2018; von Moltke et al., 2016). Yet, the interaction between intestinal tuft cells and other mucosal immune cell populations is not fully understood.

The importance of tuft cells in mucosal homeostasis is highlighted in several human gastrointestinal diseases. Indeed, tuft cell numbers are diminished in inflammatory intestinal diseases, such as ulcerative colitis and duodenitis (Huh et al., 2020; Kjaergaard et al., 2021). Interestingly, in some cases, such diseases have been ameliorated upon tuft cell restoration, as seen in a mouse model with Crohn's‐like ileitis (Banerjee et al., 2020). Differentiation of intestinal tuft cells may serve an important role in mucosal regeneration, while their depletion may contribute to the malabsorption seen in patients suffering from celiac disease or microvillus inclusion disease (MVID), consistent with our observations in Myosin VB knockout mice, which model MVID phenotypes (Huh et al., 2020; Kaji et al., 2021; Kalashyan et al., 2023). Considering the role tuft cells play in chemosensation, loss may directly induce nutrient malabsorption. To test this hypothesis, this study evaluated changes in intestinal absorptive function, secretory cell lineages, and mucosal immune cell populations when tuft cells are acutely deleted in a tamoxifen‐induced mouse model.

## 2 MATERIALS AND METHODS

### 2.1 Mice

The Institutional Animal Care and Use Committee (IACUC) of Vanderbilt University Medical Center, Nashville, TN, USA approved all experimental procedures and animal care (M2000104).

Frozen sperm from *DCLK1‐CreER* ^*T2‐IRES‐GFP*^ mice (Nakanishi et al., 2013) was a gift from Dr. Hiroshi Seno at Kyoto University and used for in vitro fertilization at the Vanderbilt Genome Editing Resource. After backcrossing with wild‐type C57Bl6/J (Strain #000664, Jackson Laboratory), *DCLK1‐CreER* ^*T2‐IRES‐GFP*^ mice were crossbred with ROSA26‐DTA mice (Strain #009669, Jackson Laboratory). *DCLK1‐CreER* ^*T2‐IRES‐GFP*^ and *DTA* alleles were maintained as heterozygous on C57Bl6/J to obtain *DCLK1‐CreER* ^*T2‐IRES‐GFP/+*^;*Rosa26‐DTA/+* (referred to as DCLK1‐DTA) and control mice (*DCLK1‐CreER* ^*T2‐IRES‐GFP/+*^ and Rosa‐DTA/+) from the same litters. Breeders were fed a breeder chow diet (Cat# 5012; LabDiet) ad libitum.

Post‐weaned mice were group‐housed under a 12 light/12 dark cycle and received a standard chow diet (Cat# 5010; LabDiet) and water ad libitum. At 7–9 weeks of age, both male and female DCLK1‐DTA mice and littermate controls received a single dose of tamoxifen citrate (80 mg/kg) by intraperitoneal (IP) injection (day 0). Body weight changes were monitored daily. On days 2, 4, or 7, mice were euthanized by CO_2_ inhalation and cervical dislocation, and the duodenum (0–8 cm from the pyloric ring), jejunum (8 cm following the duodenum), ileum (distal 8 cm from the ileocecal junction), and colon were collected. These intestinal segments were cut open along the mesenteric border, luminal contents were washed out, and tissues were fixed in 10% neutral buffered formalin (NBF) between filter papers overnight. Each segment was rolled along its proximal to distal axis (Swiss roll) with pieces of Parafilm® and fixed in NBF for 24 more hours. Fixed tissues were then embedded in paraffin.

Germline POU2F3 knockout mice, a generous gift from Dr. Ichiro Matsumoto (Monell Chemical Senses Center), and littermate controls were maintained on a CD1 background by the DelGiorno lab at Vanderbilt University (IACUC No. M2000077). At the age of 8–12 weeks, mice were euthanized by CO_2_ inhalation and cervical dislocation, and small intestinal tissues were harvested. Tissues were fixed as described above, and paraffin sections were generated as a comparison to DCLK1‐DTA mice.

### 2.2 Portal blood analysis

Blood samples were collected from the portal vein by EDTA syringes, centrifuged at 4°C, and the plasma was stored at −80°C until use. Portal hexose and lactate concentrations were analysed at the Vanderbilt Mass Spectrometry Core. Briefly, 20 μL aliquots of thawed plasma were spiked with lactic acid‐^13^C_3_, extracted with acetonitrile/methanol (2:1), and derivatized with the reagent dansyl hydrazine and the carboxyl activating agent 1‐Ethyl‐3‐(3‐dimethylaminopropyl)carbodiimide (EDC). Under these conditions, lactic acid and hexoses are converted to their corresponding hydrazide and hydrazone derivatives, respectively. Quantification was based on selected reaction monitoring (SRM) detection using an Acquity ultrahigh‐performance liquid chromatography system (Waters Corp, Milford, MA) interfaced to a TSQ Quantum triple‐stage quadrupole mass spectrometer (Thermo Fisher Scientific, Waltham, MA). The following SRM transitions were monitored using a scan time of 25 ms and a Q3 scan width of 2.0 m/z: lactic acid: m/z 338 → 171, CE 27; lactic acid‐^13^C_3_: m/z 341 → 171, CE 27; hexoses: m/z 428 → 236, CE 24. Data acquisition and quantitative spectral analysis were conducted using Thermo‐Finnigan Xcalibur version 2.0.7 SP1 and Thermo‐Finnigan LCQuan version 2.7, respectively. Calibration curves were constructed by plotting peak area ratios (analyte/internal standard) against analyte concentrations for a series of standards ranging from 50 pmol to 1000 nmol analyte. Electrospray ionization source parameters were tuned and optimized using authentic lactic acid and glucose reference standards derivatized with dansyl hydrazine and EDC.

### 2.3 IL‐25 and IL‐13 treatment

Recombinant Mouse IL‐25 (IL17E) (1399‐IL, R&D Systems, Minneapolis, MN) and Mouse IL13 (413‐ML) proteins were reconstituted in filtered PBS and stored at −80°C until use. Following tamoxifen injection, DCLK1‐DTA mice were given intraperitoneal injections of IL‐25 at 0.5 μg/mouse (von Moltke et al., 2016) or IL‐13 at 2 μg/mouse/day (Manocha et al., 2013). An equal amount of PBS was injected into a vehicle control group of mice.

### 2.4 Immunofluorescence staining and imaging

Paraffin sections were cut at a 4‐μm thickness at the Vanderbilt Translational Pathology Shared Resources (TPSR). Antigen retrieval was performed using a 10 mM sodium citrate buffer containing 0.05% Tween 20 (pH 6) in a pressure cooker for 15 min following deparaffinization and rehydration. After cooling down, slides were rinsed in 1X PBS and blocked with Dako Protein block serum‐free solution (X0909, Agilent) for 1 h at room temperature (r/t) or overnight at 4°C. The primary antibodies listed in Table 1 were diluted in Dako diluent with a background‐reducing compound (S3022) and incubated with the pre‐blocked slides overnight at 4°C. Some primary antibodies were pre‐conjugated with Alexa Fluor 555 using Zenon™ Rabbit IgG Labeling Kits (Z25305, Invitrogen). Corresponding secondary antibodies conjugated with fluorescence (Table 2) were diluted in Dako diluent (S0809) and incubated for 1 h at r/t. The stained slides were rinsed in 1X PBS, counterstained with Hoechst 33342, and cover slips were mounted with ProLong Gold Antifade Mountant (Cat# P36934; Thermofisher Scientific). Immunofluorescence was visualized using 10X or 20X objective lenses on a Zeiss Axio Imager M2 with Apotome 3 and the Zen 3.5 software (Curl Zeiss Microscopy, LLC, White Plains NY). Whole slide images were taken by a 20X objective on an Aperio Versa 200 (Leica) in the Digital Histology Shared Resource (DHSR).

**TABLE 1:** Primary antibody list.

Target	Host	Conjugation	Source	Catalog No.	Dilution
5‐HT	Rat mono	Dylight‐488	Novus	NB100‐65037G	1:100
AcTubulin	Mouse mono	Unconjugated	Sigma	T6793	1:200
Beta‐Catenin	Mouse mono	Dylight‐550	Novus	NBP1‐54467R	1:100
CD3	Rabbit poly	Unconjugated	Abcam	ab‐5690	1:1000
ChAT	Goat	Unconjugated	Millipore	AB144P	1:50
CHGA	Rabbit poly	Unconjugated	Immunostar	ID: 20085	1:2000
CK‐19	Rat mono	Unconjugated	Sigma	MABT913	1:250
c‐Kit	Goat	Unconjugated	R&D	AF1356	1:100
CLDN15	Rabbit poly	Unconjugated	ThermoFisher	38–9200	1:120
Dcamlk1	Rabbit mono	Alexa Fluor‐647	Abcam	ab202755	1:2000
EpCAM	Rabbit mono	Alexa Fluor‐488	Abcam	ab237384	1:200
Gamma‐Actin	Mouse mono	Alexa Fluor‐488	Santa Cruz	sc‐65,638 AF488	1:100
Gamma‐Actin	Mouse mono	Alexa Fluor‐647	Santa Cruz	sc‐65,638 AF647	1:100
Gamma‐Actin	Mouse mono	Alexa Fluor‐790	Santa Cruz	sc‐65,638 AF790	1:50
GATA3	Rabbit mono	Unconjugated	Abcam	ab199428	1:500
GFP	Rabbit poly	DyLight‐488	Novus	NB600‐308G	1:200
GIP	Rabbit poly	Unconjugated	Novus	NBP3‐04865	1:100
Ki67	Rat mono	Unconjugated	BioLegend	652,402	1:100
Ki67	Rabbit	Alexa Fluor‐488	Cell Signaling	11882S	1:100
LAMP1	Rat mono	Unconjugated	Santa Cruz	sc‐19,992	1:200
Lysozyme	Rabbit poly	Alexa Fluor‐750	Novus	NBP2‐61118AF750	1:100
MCPT1	Sheep	Unconjugated	R&D	AF5146	1:200
MMP‐7	Rabbit mono	Unconjugated	Cell Signaling	3801 T	1:100
Pou2F3	Rabbit poly	Unconjugated	Atlas (Sigma)	HPA019652	1:100
SGLT1	Rabbit	Unconjugated	Novus	NBP2‐38748	1:200
Somatostatin	Mouse mono	eFluor™ 660	eBioscience™	50–9751‐82	1:50
TFF3	Mouse mono	eFluor™ 660	Invitrogen	50–4758‐82	1:100
Villin	Mouse mono	Alexa Fluor‐488	Santa Cruz	sc‐58,897 AF488	1:50

**TABLE 2:** Secondary antibody list. All items were purchased from the Jackson ImmunoResearch Laboratories, Inc. (West Grove, PA).

Target	Host	Conjugation	Catalog No.	Dilution
Rabbit IgG (H + L)	Donkey	Cy™3	711–165‐152	1:400
Rabbit IgG (H + L)	Donkey	Cy™5	711–175‐152	1:400
Rat IgG (H + L)	Donkey	Cy™2	712–225‐153	1:400
Rat IgG (H + L)	Donkey	Cy™3	712–165‐153	1:400
Sheep IgG (H + L)	Donkey	Alexa Fluor® 594	713–585‐003	1:400

### 2.5 Immunostaining for Claudin‐15

Small pieces of mouse jejunum were washed in PBS to remove luminal contents and immediately frozen with OCT compound. Cryosections (15‐μm‐thin) were fixed in methanol for 10 min at −20°C. The tissue sections were pre‐incubated with PBS including normal donkey serum (10%), Triton X‐100 (0.1%), and bovine serum albumin (1%) for 1 h to block non‐specific antibody interactions. Rabbit anti‐CLDN15 (38–9200, ThermoFisher) antibody was diluted in the blocking solution and incubated on sections for 1 h at room temperature. After rinsing slides in PBS, the sections were incubated with Cy3‐conjugated donkey anti‐rabbit IgG and Hoechst 33342 diluted in PBS for 1 h at room temperature. Fluorescence was visualized using a 20X objective on the Zeiss Axio Imager M2 with Apotome.

### 2.6 Whole‐mount immunofluorescence imaging in gallbladder

Mouse gallbladders were isolated from the liver, cut open to flat sheets, and fixed in NBF between filter papers. Small pieces of gallbladder were rinsed and pre‐blocked in PBS containing 5% normal donkey serum and 0.3% Triton X‐100. Primary antibodies were diluted in the fresh blocking buffer and incubated with tissues for 2 days at 4°C. After rinsing tissues with 0.3% Triton X‐100/PBS (TBS), secondary antibodies and Hoechst 33342 diluted in TBS were incubated for 2 h at room temperature. Fluorescence was imaged using 20X and 60X objective lenses on a Nikon Ti‐E microscope with an A1R laser scanning confocal system and NIS‐Elements AR 5.30.05 software (Nikon Instruments Inc., Melville, NY).

### 2.7 Measurements of tissue architecture

The length of small intestinal crypts and villi and the height of colonic mucosa were measured on whole‐slide images of immunostained tissues for Ki67 and ACTG1 using Aperio ImageScope software (Leica, Wetzlar, Germany). A minimum of 10 regions was measured and averaged for each mouse.

### 2.8 Digital image analysis

The digital analysis was conducted at the VUMC Digital Histology Shared Resource. Utilizing whole‐slide fluorescence images, individual tissue segments were extracted, and machine learning was performed to generate probability maps of all intestinal tissues and antibody signals, as well as tissue folds and debris (Ilastik) (Berg et al., 2019). Mucosal architecture was divided into epithelial and non‐epithelial (mesenchymal and muscle) tissue, and non‐tissue area (debris and glass). These probability maps were used in combination with the original 3–5 color images to segment individual cells based on their nuclei and combined membrane marker signals using in‐house coded scripts (MatLab) (The MathWorks Inc, 2022). The scripts were optimized to quantify the various populations of immunofluorescence‐positive (^+^) cells, including CHGA^+^, CCK^+^, 5‐HT^+^, GIP^+^, SST^+^, LYZ^+^, MMP7^+^, TFF3^+^, DCLK1^+^, or GATA3^+^. These marker^+^ cells were identified and filtered by their size, location, and presence of a detectable nucleus. The percent of each immunopositive cell population was calculated per total number of epithelial cells in whole Swiss rolls.

### 2.9 Ussing chamber experiments

Mucosal‐submucosal preparations were obtained from the jejunum and mounted in sliders with an aperture = 0.3 cm^2^ (Physiologic Instruments, Leno, NV) as described previously (Kaji et al., 2020). Gallbladders were cutopen and mounted in sliders with an aperture = 0.031 cm^2^. Luminal and serosal surfaces of the tissue were bathed in 4 mL Krebs‐Ringer solution (117 mM NaCl, 4.7 mM KCl, 1.2 mM MgCl_2_, 2.5 mM CaCl_2_, 1.2 mM NaH_2_PO_4_, 25 mM NaHCO_3_, 11 Mm glucose) and maintained at 37°C using a water‐recirculating heating system. Indomethacin (10 μM) was added to the serosal bath for the jejunum to suppress prostaglandin‐stimulated secretion at baseline. The solution was continuously bubbled with a gas mixture of 95% O_2_ and 5% CO_2_ to maintain pH at 7.4. Short‐circuit current (*I*_sc_) was continuously recorded under voltage clamp conditions at zero potential difference by the DataQ system (Physiologic Instruments). Transmucosal resistance (*R*t) was determined by changing the clamped voltage automatically at ±3 mV for 20 ms every 2 s. An increase in *I*_sc_ indicates luminal‐to‐serosal current flow, for example, anion secretion or cation absorption. Tissues were stabilized for 20 min before the effects of drugs were investigated. SGLT1 activity was represented by phlorizin (0.1 mM)‐sensitive *I*_sc_. Cl^−^ secretion was measured by *I*_sc_ peaks after carbachol (10 μM) and forskolin (10 μM). CFTR activity was determined using the CFTR inhibitor, (R)‐BPO‐27 (10 μM). DMSO <0.3% in the bathing solution did not affect the *I*_sc_ or *R*_t_.

### 2.10 RNA extraction and quantitative PCR

Total RNA was prepared from scraped mucosa of the jejunum by using TRIzol™ Reagent (Cat# 15596018; Thermo Fisher Scientific, Waltham, MA). After reverse transcription of the RNA using SuperScript III First‐strand Synthesis System for RT‐PCR (Cat# 18080051; Thermo Fisher Scientific), cDNA was used as a template for PCR. Real‐time quantitative PCR was performed utilizing SsoAdvanced Universal SYBR Green Supermix (Cat# 1725271) with a CFX96 Real‐Time System (Bio‐Rad Laboratories). Previously published primer pairs used were listed in Table 3. Target gene expression was calculated as relative values by the ΔΔCt method.

**TABLE 3:** Primer sequences for qRT‐PCR.

GENE	Forward seq (5′‐3′)	Reverse seq (5′‐3′)	Ref
TNFα	CTGAACTTCGGGGTGATCGG	GGCTTGTCACTCGAATTTTGAGA	Huang et al. (2018)
IL‐10	CTTTGCTATGGTGTCCTTTCA	AAGACCCATGAGTTTCTTCAC	Matsumoto et al. (2011)
NOS2	TAAATCTCTCTCCTCTCCTCC	TTTTTCTCTGCTCTCAGCTCC	Yi et al. (2019)
Reg3beta	GGCTTCATTCTTGTCCTCCA	ATCCACCTCCATTGGGTTCT	McKinley et al. (2017)
Reg3delta	ACTGTGTTGCCTGATGTCCC	TAGCCCAGGTCTGTGGTTCC	McKinley et al. (2017)
Reg3gamma	GTATGATGCAGATATGGCCTG	ATATTGGCCACTGTTACCAC	Billipp et al. (2024)
Duox1	CACCAGGAACGGATTGTTCT	CCTGCAAGCCAAAAGAAGAC	Schutz et al. (2019)
Duox2	TGCAACAGCTACTGGATTCG	ATCCTGTCCTCCAGCTCTGA	Schutz et al. (2019)
IL‐13	TGGGTGACTGCAGTCCTGGCT	GTTGCTTTGTGTAGCTGAGCA	Gerbe et al. (2011)
Lyz1	ATGGCTACCGTGGTGTCAAG	CGGTCTCCACGGTTGTAGTT	Ndjim et al. (2024)
Pou2f3	ACCCATCTACAACTCCCGGC	CCAGGGGAACAGGATGACGT	Ndjim et al. (2024)
Nlrp1b	CAACAAGACTTGAACACAACGAG	CTCTCAATGACTGTGCTGGGTA	Bezencon et al. (2008)
Ldhb	AGTCTCCCGTGCATCCTCAA	AGGGTGTCCGCACTCTTCCT	Moran et al. (2021)
Wnt5a	CAC GCT ATA CCA ACT CCT CTG C	AAT ATT CCA ATG GGC TTC TTC ATG GC	von Moltke et al. (2016)
ChAT	AGTAAGGCTATGGGATTCAATC	AGTTCACCTTGATGCCGTTC	Gerbe et al. (2016)

### 2.11 Chemicals

Tamoxifen citrate (USP, Spectrum™ Chemical 18–607‐202) and 10x PBS were purchased from Thermo Fisher Scientific (Hanover Park, IL), and other chemicals were from Sigma Aldrich (St. Louis, MO) unless specified. Tamoxifen citrate (20 mg/mL) was dissolved in 10% DMSO and 90% corn oil and warmed in a water bath at 65°C. Indomethacin (Cat# I7378) was dissolved in 100% ethanol. Forskolin was purchased from Cayman Chemical (Cat# 11018, Ann Arbor, MI), (R)‐BPO‐27 (Cat# HY‐19778), and CaCCinh A‐01 (Cat# HY‐100611) were from MedChemExpress (Monmouth Junction, NJ) and were dissolved in DMSO to prepare 1000x stocks.

### 2.12 Statistics

Statistical differences were determined using GraphPad Prism 8. Significant *p* values of ≤ 0.05 were indicated in red in each graph. Each datapoint indicates the result of an individual mouse, and bars indicate mean ± S.D. unless specified in the figure legend. The test used in each analysis is described in the figure legends.

## 3 RESULTS

### 3.1 Diphtheria toxin‐induced acute tuft cell loss decreased nutrient absorption in the small intestine

**FIGURE 1:**
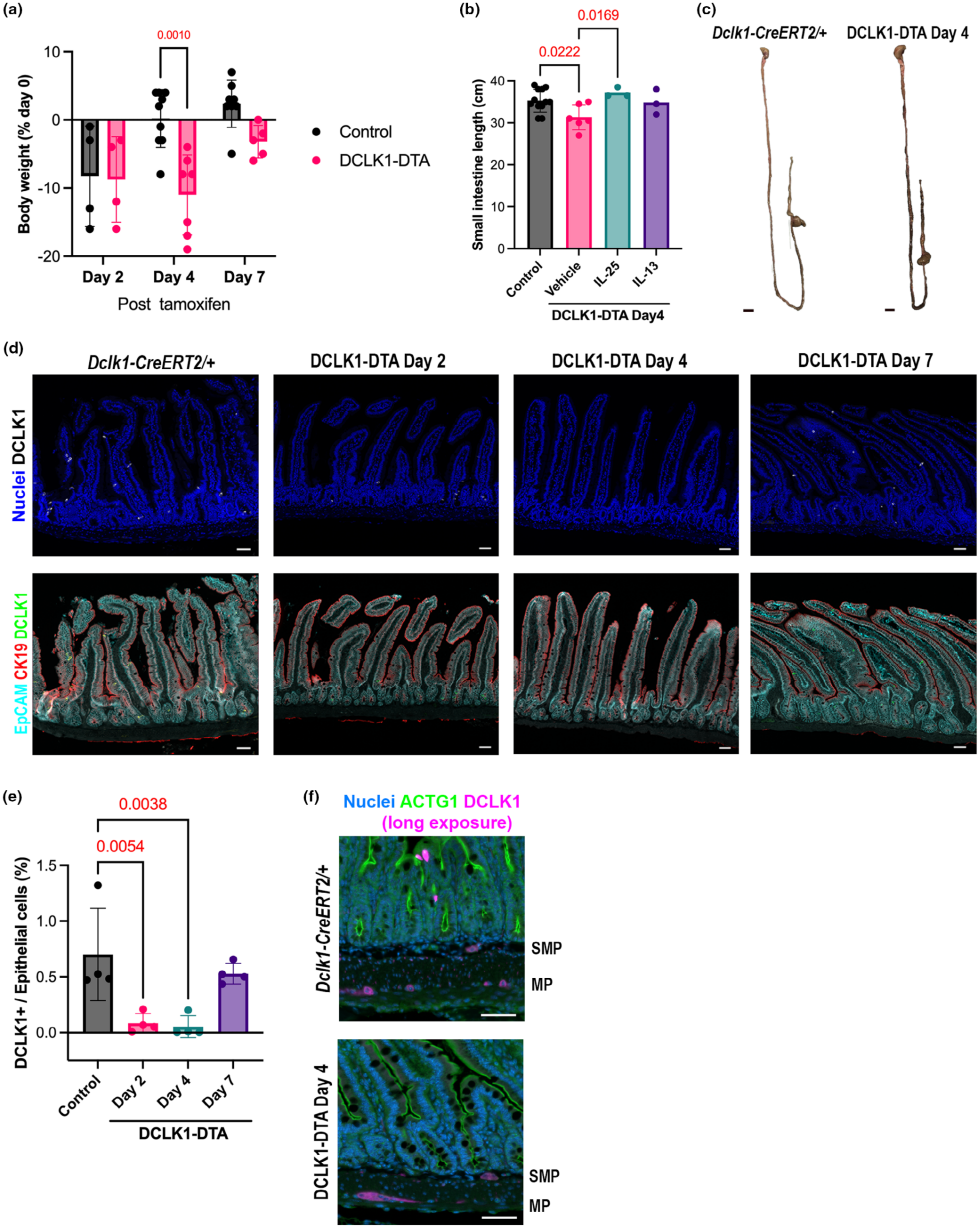
Tuft cell deletion in mouse small intestine reduces body weight and intestinal length. (a) Significant body weight loss is observed only on day 4 post tamoxifen treatment in DCLK1‐DTA mice compared to control mice by two‐way ANOVA with Tukey's test. (b) The small intestine is significantly shortened on day 4 in DCLK1‐DTA mice as compared to control mice and is restored upon IL‐25 supplementation (ANOVA with Dunnett's test). (c) Representative images of the whole GI tract from control mice and DCLK1‐DTA day 4 mice. Scale bars = 1 cm. (d) Immunostaining for DCLK1 in jejunum sections. Epithelial markers, EpCAM, and CK19, are co‐stained to conduct cell counting by imaging analysis scripts. Compared to healthy control mice, DCLK1‐DTA mice demonstrate transient decreases in tuft cells after a tamoxifen injection. Scale bars = 50 μm. (e) Quantification of DCLK1+ cells per total epithelial cell numbers. More than 95% deletion occurs in DCLK1‐DTA mice on day 4 (ANOVA with Dunnett's test). (f) Immunostaining for DCLK1 in submucosal plexus (SMP) and myenteric plexus (MP) of jejunum. Epithelial gamma‐Actin (ACTG1) and nuclei are counterstained. The stained signals in neurons are weaker than those of tuft cells, but similar in level between control and DCLK1‐DTA tissues. Scale bars = 50 μm.

The impact of acute tuft cell deletion on intestinal absorptive function has not been fully explored. We investigated the effects of transient DCLK1^+^ tuft cell loss on epithelial cell differentiation and nutrient absorptive ability in the small intestine. After a single dose of tamoxifen, DCLK1‐DTA mice showed a decrease in body weight within 4 days and recovered to initial body weight by day 7 (Figure 1a). Tamoxifen citrate slightly decreased body weight on day 2 independent of mouse genotype or sex, likely due to nonspecific toxicity. Body weight loss in DCLK1‐DTA mice on day 4 was significant as compared to tamoxifen‐treated control littermates. Consistent with body weight loss, the small intestinal length, but not colonic length, was significantly shorter in DCLK1‐DTA mice as compared with those of controls at day 4 post‐tamoxifen (Figure 1b,c). The shortening of intestinal length was restored upon the administration of IL‐25 to DCLK1‐DTA mice (Figure 1b). On day 4 post‐tamoxifen, body weight changes were not significant after treatment with IL‐25 (−0.003 ± 1.8%). Immunostaining for DCLK1 in the small intestines revealed that intestinal tuft cell numbers were decreased in DCLK1‐DTA mice by 85% on day 2, largely abolished on day 4, and completely recovered on day 7, consistent with their body weight changes (Figure 1d,e). DCLK1 expression in enteric neurons, however, was not altered by a single tamoxifen injection in DCLK1‐DTA mice (Figure 1f). The correlation between tuft cell numbers and small intestinal length suggests that acute deletion of tuft cells may influence mucosal cell proliferation; therefore, we explored this as a possibility in our mouse model.

**FIGURE 2:**
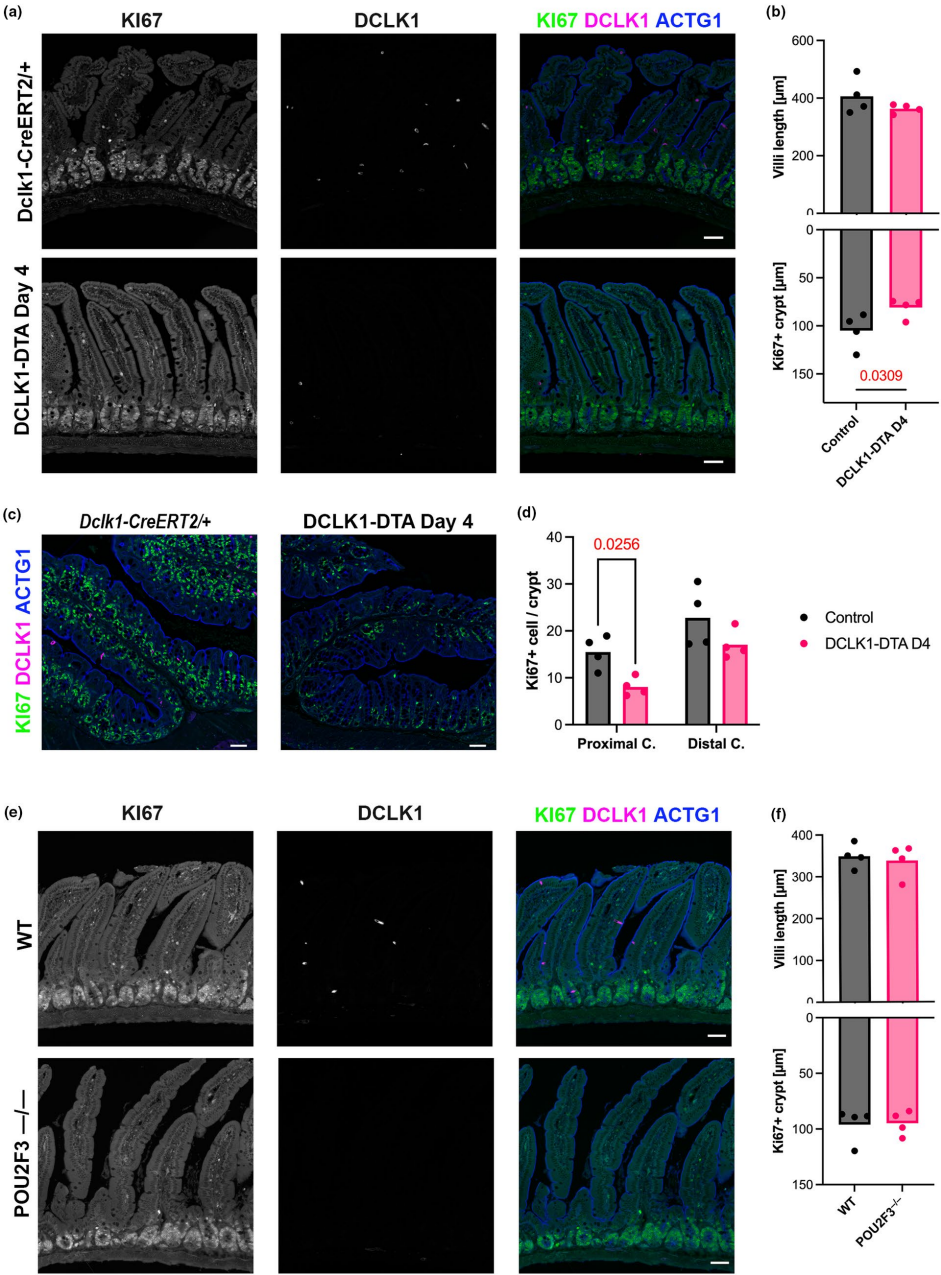
Reduced intestinal proliferation in tuft cell deleted mice. (a and c) Immunostaining for a proliferative cell marker, Ki67 (green), an epithelial‐dominate Actin, gamma‐Actin (ACTG1, blue), and DCLK1 (magenta) in the duodenum (a) and proximal colon (c) of DCLK1‐DTA mice at Day 4. (b and f) Ki67+ length in the crypts and villus height are measured in 10 regions of jejunal Swiss roll of each mouse. Each datapoint indicates an average value of individual mouse, and the bars represent mean values of group. Decreased crypt proliferation is characterized in DCLK1‐DTA mice, but not POU2F3^−/−^ mice, compared to littermate control mice (unpaired *t*‐test). (d) Ki67+ nuclei are counted per vertical section of crypt in proximal and distal colons. Significant reduction of proliferative cells is identified in the proximal, but not distal colon, of DCLK1‐DTA mice by two‐way ANOVA with LSD test. (e) POU2F3^―/―^ mice show no significant difference in Ki67+ crypt length or villus structures when compared to control littermates. Scale bars = 50 μm.

Immunostaining for a proliferation marker, Ki67, revealed a decrease in the length of the Ki67^+^ zone of the intestinal crypts in DCLK1‐DTA mice on day 4, whereas villus length was not significantly affected between DCLK1‐DTA and control tissues (Figure 2a,b). This significant decrease in Ki67^+^ cells was also identified in the proximal colon, but not in the distal colon (Figure 2c,d). To evaluate whether the effect of tuft cell loss on proliferative cells is consistent in a constitutive mouse model of tuft cell ablation, we performed immunostaining for Ki67 in whole‐body *Pou2f3* knockout mice (POU2F3^―/―^), which lack the Pou domain class 2 transcription factor 3, the master regulator transcription factor for tuft cell development. The lengths of the Ki67+ zone and villus lengths were comparable between POU2F3^―/―^ and wild‐type (WT) littermates (Figure 2e,f). Body weight of POU2F3^―/―^ mice and age‐matched littermate controls had no significant difference (data not shown). These observations indicate that acute tuft cell deletion affects mucosal homeostasis and nutrient absorption of the small intestine and proximal colon, whereas germline POU2F3 ablation on an outbred mouse background may possess compensation pathways to maintain epithelial proliferation. The DCLK1‐DTA mouse tissues on day 4 (referred to as DCLK1‐DTA) were then analysed in subsequent experiments.

**FIGURE 3:**
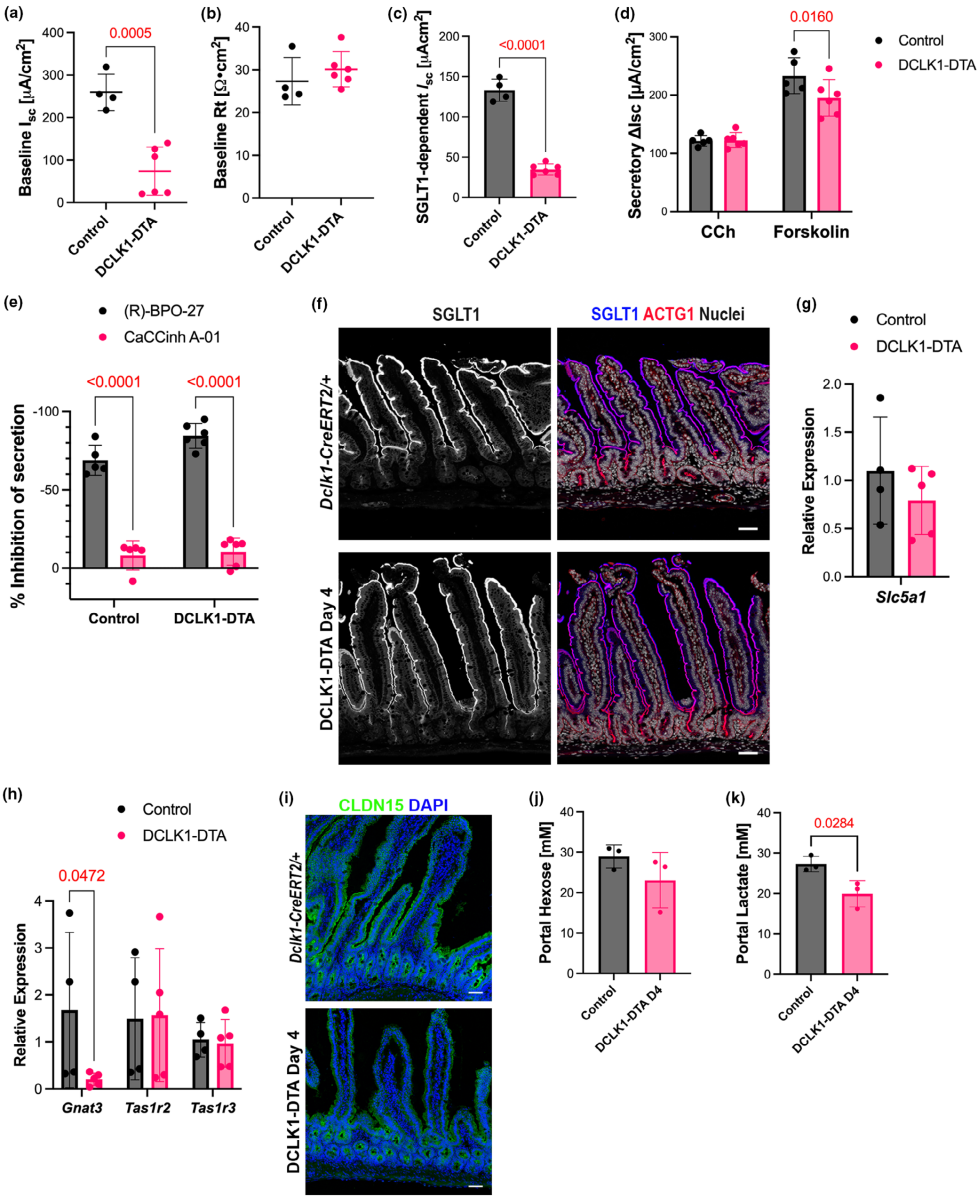
SGLT1 and CFTR functions are reduced in DCLK1‐DTA mouse jejunum at Day 4. (a–e) Epithelial ion transport measurements in jejunum utilizing Ussing chambers. Baseline short‐circuit current (Isc) (a) is significantly lower in DCLK1‐DTA day 4 mice than in that of control, whereas baseline transmural resistance (Rt) (b) is comparable. DCLK1‐DTA mice demonstrate significantly lower levels of SGLT1‐mediated absorption (c) and forskolin‐stimulated, but not carbachol (CCh)‐mediated secretion (d), compared to control. Dependency on CFTR and CaCC of the stimulated secretion (e) have no statistical difference between genotypes. Statistical significance is detected by unpaired t‐test (a–c) or two‐way ANOVA with Tukey's test (d and e). (f) Immunostaining for SGLT1 (blue) and ACTG1 (red) in jejunum. SGLT1 is colocalized with ACTG1 on the apical brush border of villus epithelial cells in DCLK1‐DTA and control mice. (g) Relative expression of mRNA calculated with ΔΔCt method. No significant difference is detected by Mann–Whitney test in Slc5a1 (SGLT1) expression of jejunum mucosa between DCLK1‐DTA mice compared to control. Each datapoint indicates an individual mouse. (h) Relative expression levels of glucose sensors. Gnat3 (Gustducin) is significantly reduced in DCLK1‐DTA day 4 mice compared to control (two‐way ANOVA with LSD test). (i) Immunostaining for Claudin‐15 (CLDN15, green) in jejunum. CLDN15 is localized on the tight junctions of epithelial cells and no discernible difference was identified between DCLK1‐DTA and control mice. Scale bars = 50 μm. (j–k) Concentrations of hexose and lactate in portal blood are determined by mass‐spectrometry (unpaired *t*‐test).

We investigated whether the absorptive function of the intestine was affected by acute tuft cell deletion. Epithelial transporter function was assessed for the sodium‐dependent glucose transporter 1 (SGLT1), cystic fibrosis transmembrane regulator (CFTR), and Calcium‐activated Chloride Channels (CaCCs) in mouse jejunum utilizing Ussing chambers. Baseline short‐circuit current (*I*_sc_) in steady state was significantly lower in DCLK1‐DTA mice as compared to control mice (Figure 3a). In contrast, transmural mucosal resistance (Rt) was comparable at steady state, indicating an intact mucosal barrier function (Figure 3b). The contributions of SGLT1, CFTR, and CaCCs to the mucosal generated *I*_sc_ were evaluated by using selective inhibitors against each transporter. SGLT1‐mediated absorptive current was induced by 11 mM glucose in the luminal buffer and was inhibited by phlorizin. This absorptive current was significantly reduced in DCLK1‐DTA mice by approximately 80% as compared to control mice (Figure 3c). Chloride secretory responses were assessed using carbachol (CCh) and forskolin, which increase intracellular calcium and cAMP, respectively. While CCh‐induced transient chloride secretion was comparable between DCLK1‐DTA and control mice jejunum, forskolin‐stimulated secretory current was decreased by 10% in DCLK1‐DTA mice as compared to controls (Figure 3d). Subsequently, CFTR and CaCC dependence in the secretory state was evaluated by adding a CFTR inhibitor, R‐BPO‐27, followed by a CaCC inhibitor, CaCCinh A‐01. The secretory state *I*_sc_ was approximately 80% dependent on CFTR in both control and DCLK1‐DTA mice, which was significantly larger than the CaCC‐dependent portion (Figure 3e). The observed decreases in sodium absorption and chloride secretion suggest a significant change in nutrient absorption capability in the small intestine following tuft cell depletion.

Next, the molecular machinery for glucose absorption was evaluated, including SGLT1, alpha‐gustducin, and CLDN15. In immunostained murine small intestinal sections, SGLT1 consistently colocalized with the brush border marker, gamma‐actin (ACTG1), in villus epithelial cells in both DCLK1‐DTA and control mice (Figure 3f). CLDN15 is a cation channel‐forming claudin protein involved in SGLT1 activity through sodium recycling (Tamura et al., 2011). Immunostaining for CLDN15 identified expression on the basolateral membrane of enterocytes of villi, in a pattern that is indistinguishable between DCLK1‐DTA and control mouse jejunum (Figure 3f). These immunostaining patterns indicate that acute tuft cell deletion had no effect on the localization of apical SGLT1 or basolateral CLDN15, which are important for glucose absorption. Additionally, qPCR was performed with mRNA extracted from scraped jejunal mucosa of mice for *Slc5a1*, the gene that encodes SGLT1, *Gnat3*, which encodes alpha‐gustducin, and two subunits of the sweet taste receptor, *Tas1r2* and *Tas1r3*. The transcription level of *Slc5a1* was comparable between DCLK1‐DTA and control mice (Figure 3g). *Gnat3* expression was significantly reduced in the jejunal mucosa of DCLK1‐DTA mice as compared to control, whereas *Tas1r* members were similar to controls, possibly due to the compensatory expression in non‐tuft cells (Figure 3h). Gustducin is a chemosensory molecule in taste buds shown to also be important for the sensory function of tuft cells in the small intestine (Hofer et al., 1996; Howitt et al., 2016; Margolskee et al., 2007). The observed reduction of *Gnat3* expression as a glucose sensor and reduced absorptive function of SGLT1 most likely corresponds to the acute deletion of tuft cells in DCLK1‐DTA mice.

To investigate total nutrient absorption status from the intestine, portal blood from Day 4 mice was analysed by mass spectrometry. Concentrations of hexose, including glucose, galactose, and fructose, in control and DCLK1‐DTA mice were comparable (Figure 3j). Portal lactate levels are an indicator of nutrient status and decrease under conditions of fasting (Kimura et al., 1988). DCLK1‐DTA mice had significantly decreased portal lactate concentration as compared to controls (Figure 3k).

### 3.2 Aberrant secretory cell differentiation is associated with acute tuft cell loss in the small intestine

**FIGURE 4:**
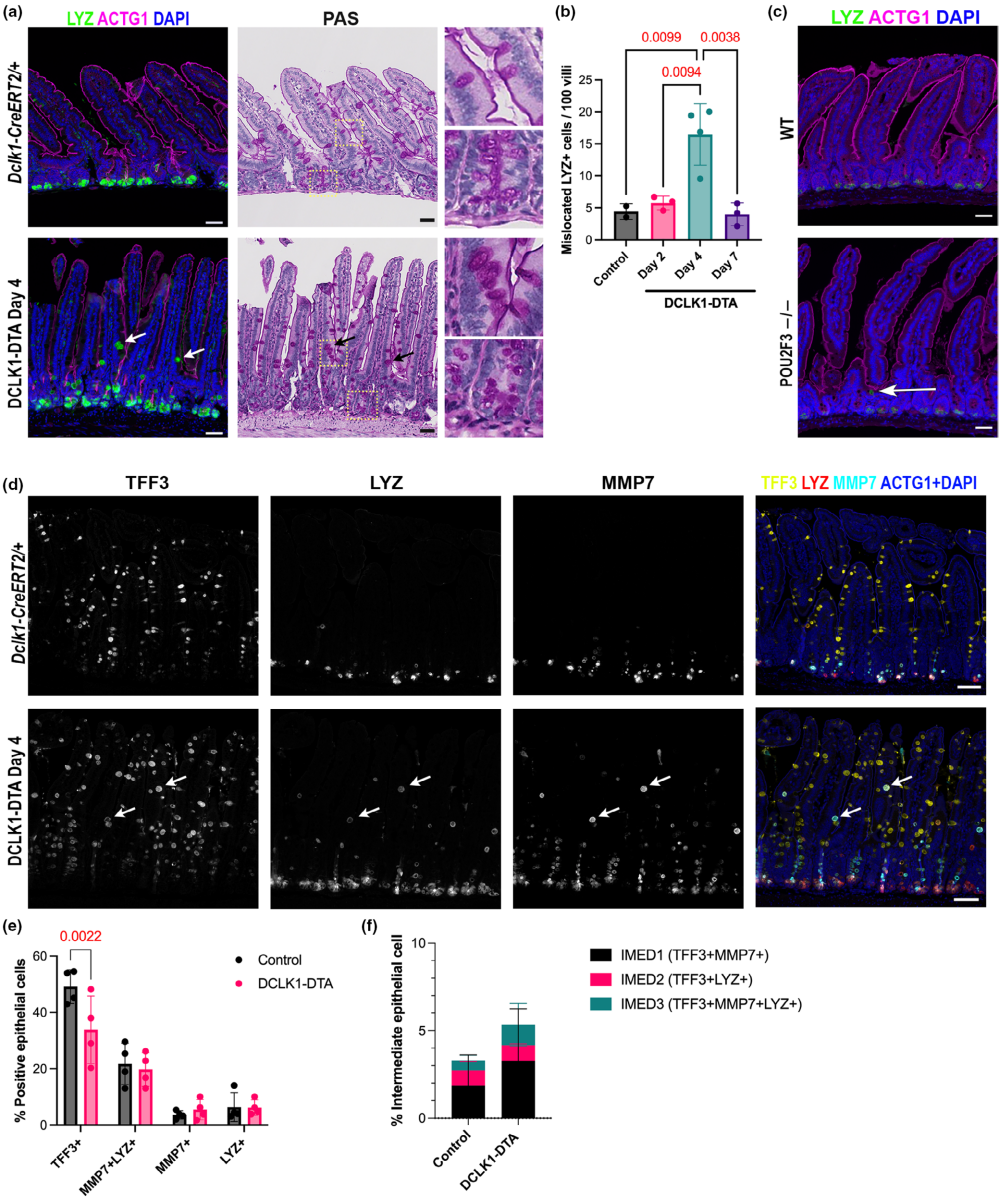
LYZ^+^ cells are Mislocalized in acute and germline tuft cell deletion models. (a) Immunostaining for LYZ (green) and ACTG1 (magenta) in jejunum. in control tissues, LYZ^+^ Paneth cells are limited to the bottom of crypts, whereas DCLK1‐DTA mice at Day 4 possess LYZ^+^ cells in upper crypt and villus regions (arrows). PAS staining was performed on the same slides after immunostaining. Insets show LYZ^−^ goblet cells and LYZ^+^ Paneth cells in control and LYZ^+^ mislocalized cells in DCLK1‐DTA mice. Scale bars = 50 μm. (b) LYZ^+^ cells localized out of the crypt bottom are counted among 100 villi of jejunum (ANOVA with Tukey's test). (c) POU2F3^―/―^ mouse tissues demonstrate mislocalized LYZ+ (green) cells out of crypts (arrow). (d) Co‐immunostaining for TFF3 (yellow), LYZ (magenta), and Zenon‐labeled MMP7 (cyan) in jejunum. ACTG1 and nuclei are shown in blue for visualizing tissue morphology. Triple‐positive cells for all markers (white arrows) are localized in villus region of DCLK1‐DTA day 4 tissues. (e, f) Digital image analysis for immunostained cell quantification in entire small intestine (two‐way ANOVA with LSD test). TFF3‐single positive cell number per all epithelial cells is significantly reduced in DCLK1‐DTA mouse small intestine (e). There was no difference in frequency of Paneth cell marker‐positive, TFF3^−^ cells. Double‐ or triple‐positive cell numbers for both goblet and Paneth cell markers are shown as intermediate (IMED) type of cells (f).

Recent evidence has shown that deletion of SOX9, an integral transcription factor for Paneth cell development, increased tuft cell frequency and activity in the small intestine (Coutry et al., 2023). Therefore, we investigated our DCLK1‐DTA model for a connection between secretory cell lineage differentiation and tuft cells. First, we performed immunostaining for lysozyme (LYZ), an established Paneth cell marker. In addition to typical Paneth cell positioning at the base of crypts, we found LYZ^+^ cells further migrated toward villus tips more frequently in DCLK1‐DTA mice than in control tissues (Figure 4a). Mislocated LYZ^+^ epithelial cells in villus regions were counted and compared between control and DCLK1‐DTA mice for all time points. LYZ^+^ cells were rarely present in the villi of control and DCLK1‐DTA murine intestines at day 2 and day 7. However, the frequency of mislocalized LYZ+ cells increased three‐ to four‐fold in DCLK1‐DTA mice on day 4, correlating with total tuft cell deletion (Figure 4b). Periodic Acid‐Schiff (PAS)‐staining on the same sections illustrated that these cells have a goblet cell‐like morphology: diffuse cellular staining as opposed to the tighter granular pattern in typical Paneth cells (Figure 4a). This result led us to hypothesize that this cell population may possess mixed characteristics of Paneth and goblet cells. Interestingly, mislocalized LYZ^+^ secretory cells were also present in the POU2F3^―/―^ mice, indicating that the formation of these cells was most likely connected to the absence of tuft cells (Figure 4c).

### 3.3 Tuft cell deletion reciprocally increases enteroendocrine cells in the small intestine

**FIGURE 5:**
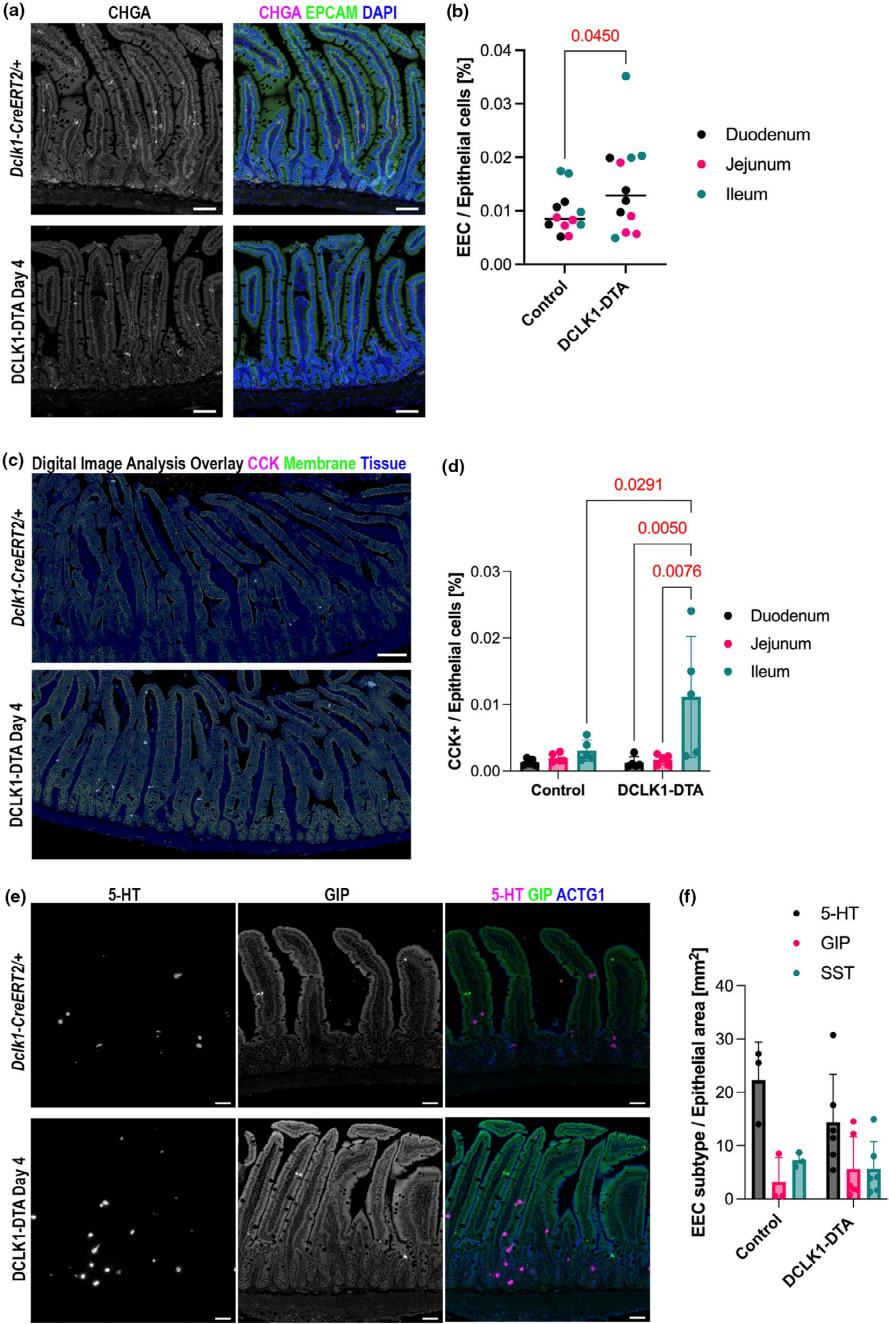
Enteroendocrine cell (EEC) numbers are increased in mouse small intestine following acute tuft cell deletion. (a) Immunostaining for a pan‐enteroendocrine cell marker, CHGA in small intestines of control and DCLK1‐DTA mice. Scale bars = 50 μm. (b) EpCAM^+^/CHGA^+^ cell (EEC) numbers per all epithelial cells are significantly increased by acute tuft cell deletion. Each datapoint indicates an individual intestinal roll, and bars indicate median (unpaired *t*‐test). *N* = 4 mice. (c) Overlay of immunostaining images for CCK and EpCAM with epithelial cell segmentation generated by digital image analysis. Light gray subjects indicate CCK^+^ (magenta) EECs in the tissue area (blue). (d) CCK+ cell frequency was significantly higher in the ileum of DCLK1‐DTA cells (two‐way ANOVA with Tukey's test). (e) Co‐immunostaining for EEC subtypes, 5‐HT (magenta), Zenon‐labeled GIP (green), an SST (not in the fields shown), with an epithelial structural marker, ACTG1 (blue). Scale bars = 50 μm. (f) Positive cell numbers for 5‐HT, GIP, or somatostatin (SST) per total epithelial cells are quantified by digital image analysis scripts. Each datapoint indicates an average value of entire small intestine in individual mouse. *N* = 3–5 mice. No statistical difference is detected by two‐way ANOVA.

To test the hypothesis that these mixed‐labeled secretory cells are present due to stalled differentiation in all secretory cell lineages, we next investigated enteroendocrine cell numbers (EECs). After acute tuft cell depletion, immunostaining was conducted for a pan‐EEC marker, chromogranin A (CHGA) (Figure 5a). The CHGA^+^ EEC population in the epithelium was determined by digital image analysis tools in the entire small intestine and revealed a significant increase in DCLK1‐DTA mice as compared to control mouse tissues (Figure 5b). We next sought to identify which EEC subpopulations increased following tuft cell loss. Cholecystokinin (CCK) has been reported to play a role in helminth clearance (Worthington et al., 2013). Immunostaining for CCK and digital image analysis were performed to quantify all CCK^+^ cells in the entire small intestine (Figure 5c). Results revealed that the CCK^+^ EEC subpopulation was significantly higher in the ileum of DCLK1‐DTA mice as compared to control mice and other intestinal segments in DCLK1‐DTA mice (Figure 5d). Other major EEC subtypes were further evaluated, including serotonin+ (5‐HT) enterochromaffin cells, gastric inhibitory peptide^+^ (GIP), and somatostatin^+^ (SST) populations (Figure 5e). Positive cells were digitally quantified, and comparable numbers of each subpopulation were detected between DCLK1‐DTA and control mice (Figure 5f). Taken together, these observations suggest a specialized epithelial cell remodeling pattern following acute tuft cell depletion in the murine small intestine.

### 3.4 Acute tuft cell deletion alters mucosal immune cell populations in the small intestine

**FIGURE 6:**
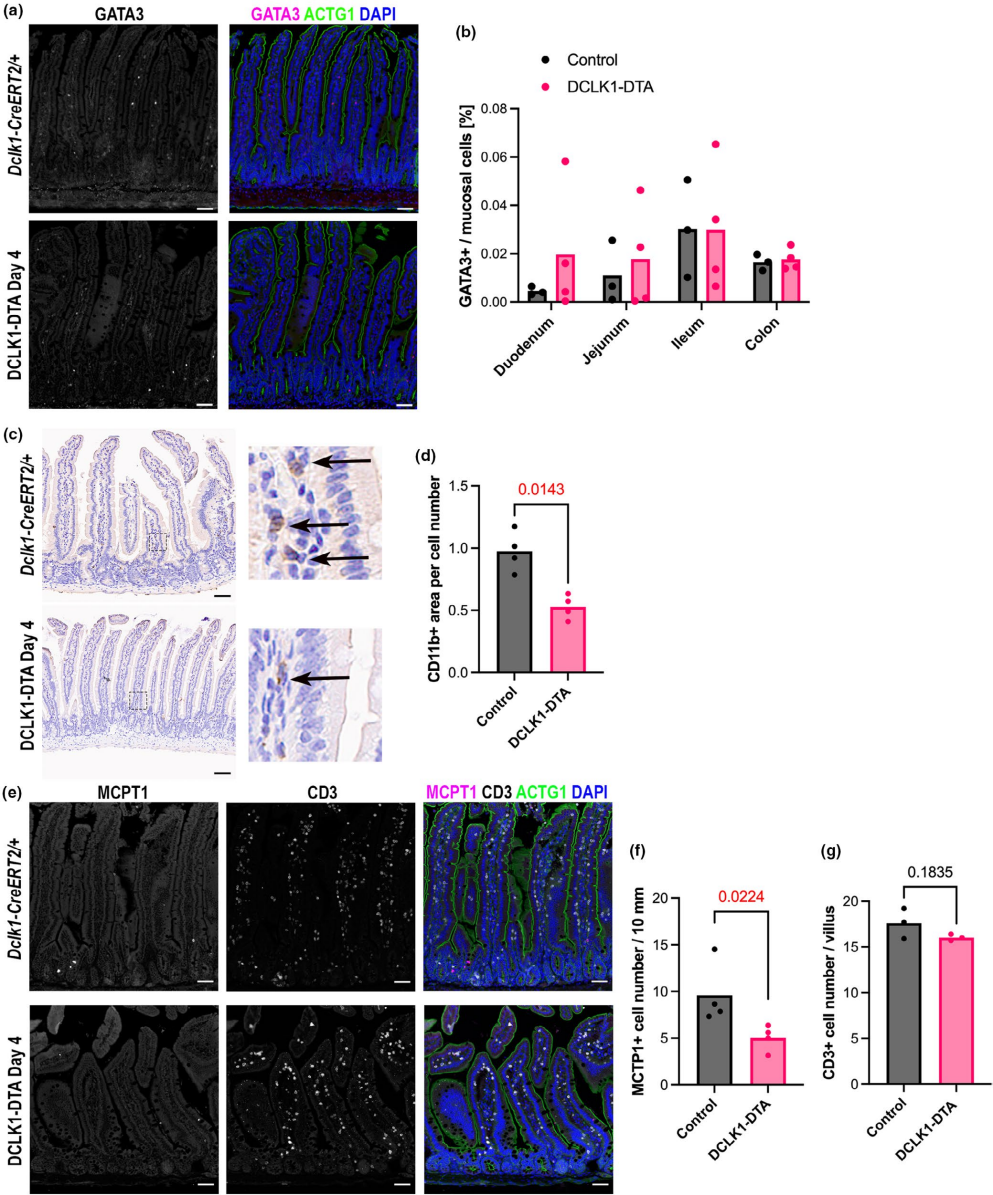
Tuft cell deletion alters mucosal immune cell populations in DCLK1‐DTA mice at Day 4. (a) Immunostaining for GATA3 (magenta), a marker for ILC2s, and ACTG1 in duodenum. GATA3 is expressed in a rare population of lamina propria cells in control and DCLK1‐DTA tissues. Scale bars = 50 μm. (b) Digital quantification of GATA3^+^ cells per total mucosal nuclei in each Swiss roll of intestine. No statistical difference is detected by two‐way ANOVA. (c) Immunohistochemistry for leukocyte marker, CD11b, in duodenum with hematoxylin counterstaining. Insets show CD11b^+^ immune cells in lamina propria (black arrows). (d) Quantification of CD11b^+^ cell area per mucosal cell nucleus number. DCLK1‐DTA mouse tissues demonstrated significantly less CD11b^+^ cells than controls (Mann–Whitney test). Each datapoint indicates an average value of >10 images of an individual mouse. (e) Immunostaining for mast cell marker, MCPT1 (magenta), a T‐cell marker, CD3, and ACTG1 in duodenum. (f) MCPT1^+^ cell numbers are significantly decreased in DCLK1‐DTA mouse duodenum compared to controls (unpaired *t*‐test). (g) CD3^+^ lymphocytes are comparable between mouse groups.

Next, we investigated whether changes occur in mucosal immune cell populations in the intestine following acute tuft cell deletion. We analysed GATA binding protein 3 (GATA3), a marker of ILC2 populations, which have been shown to respond to tuft cell activity (Cui et al., 2023). Immunostaining for GATA3 was sporadically identified in the lamina propria in both crypt and villus regions (Figure 6a). There was no significant difference in the number of GATA3+ cells in whole Swiss rolls of small intestine between genotypes (Figure 6b). Immunohistochemistry (IHC) was performed for CD11b, which is encoded by *Itgam* and is a marker of myeloid cells, including leukocytes and dendritic cells (Figure 6c). Subsequent analyses utilizing QuPath (Bankhead et al., 2017) revealed that the CD11b^+^ area was significantly lower in the jejunum of DCLK1‐DTA mice as compared to controls (Figure 6d). The number of mucosal mast cells increased in response to type 2 immune activation and tuft cell‐derived IL‐25 (Leyva‐Castillo et al., 2019) as shown by immunostaining for mast cell protease 1 (MCPT1). MCPT1^+^ populations were rare in both control and DCLK1‐DTA murine intestines and were localized in the lamina propria adjacent to the epithelial layer (Figure 6e). Their numbers were compared per 10 mm of longitudinal length of the jejunum between genotypes. We identified a 50% reduction in MCPT1^+^ mast cell counts in the DCLK1‐DTA mouse jejunum as compared to control (Figure 6f). Lastly, we immunostained for CD3 to assess the major intraepithelial T lymphocyte population, which is important for maintaining the mucosal barrier (Figure 6e). There was no significant change in localization or numbers of CD3^+^ lymphocytes following tuft cell deletion (Figure 6g).

### 3.5 Absence of inflammation and unchanged growth factor levels in the shortened small intestine following tuft cell deletion

**FIGURE 7:**
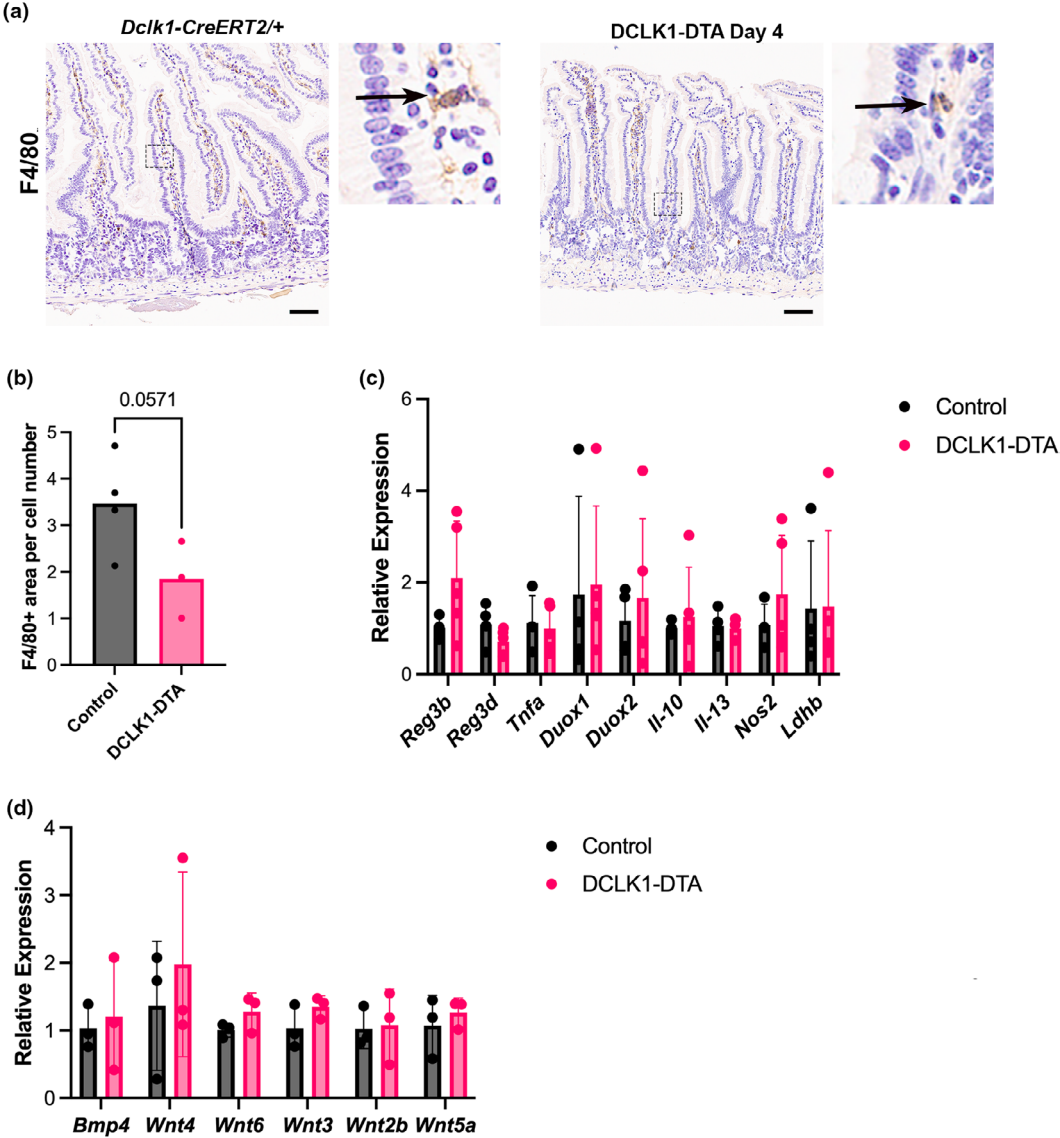
Lack of changes in proinflammatory and growth factor signaling molecules in DCLK1‐DTA mice at Day 4. (a) Immunohistochemistry for F4/80, a marker of macrophage and dendritic cells, in duodenum. Scale bars = 50 μm. (b) Quantification of F4/80^+^ cell area per mucosal cell nucleus number. No statistical difference is detected by the Mann–Whitney test. (c) Transcription levels of proinflammatory markers in scraped mucosa. No difference is detected by two‐way ANOVA. (d) Transcription levels of secretory growth factors that influence epithelial cell proliferation had no significant differences.

Small intestinal shortening is frequently coincident with mucosal inflammation (Rangan et al., 2019). However, there was no evidence of significant inflammation (based on lymphocyte infiltration) on H&E and IHC‐stained tissues (Figure 6) from DCLK1‐DTA mice. To confirm that tuft cell ablation has no impact on intestinal inflammation, we performed IHC for the macrophage/dendritic cell marker, F4/80 (also known as Adhesion G protein‐coupled receptor E1: ADGRE1). No abnormal infiltration of F4/80+ immune cells was identified in DCLK1‐DTA tissues as compared to controls (Figure 7a,b).

Proinflammatory marker genes were assessed by qRT‐PCR using RNA from scraped jejunal mucosa. Transcription levels were assessed for regeneration family members (Reg3‐beta and ‐gamma), cytokines such as tumor necrosis factor‐alpha (*Tnfa*), interleukin 10 (*Il‐10*), and interleukin 13 (*Il‐13*), inflammatory marker enzymes including lactate dehydrogenase B (*ldhb*), *Duox1* and *Duox2*, and inducible nitric oxide synthase (*Nos2*). There was no significant difference in gene expression for any marker between DCLK1‐DTA mice on day 4 versus controls, indicating that the intestinal mucosa did not initiate an inflammatory response when tuft cells were acutely deleted (Figure 7c). Due to the decreased epithelial proliferation in the DCLK1‐DTA mice, we assayed for the expression of growth factors, including Wnt family ligands and a Wnt inhibitor, Bmp4. As Wnt is associated with adult tissue regeneration (Qi et al., 2017), we sought to determine if lower levels of Wnt or higher Bmp4 could be responsible for the shorter small intestine. However, there was also no significant difference in the expression of these growth factors between DCLK1‐DTA mice and control tissues on day 4 post tamoxifen (Figure 7d).

### 3.6 Biliary tuft cells are depleted along with intestinal tuft cells in DCLK1‐DTA mice

**FIGURE 8:**
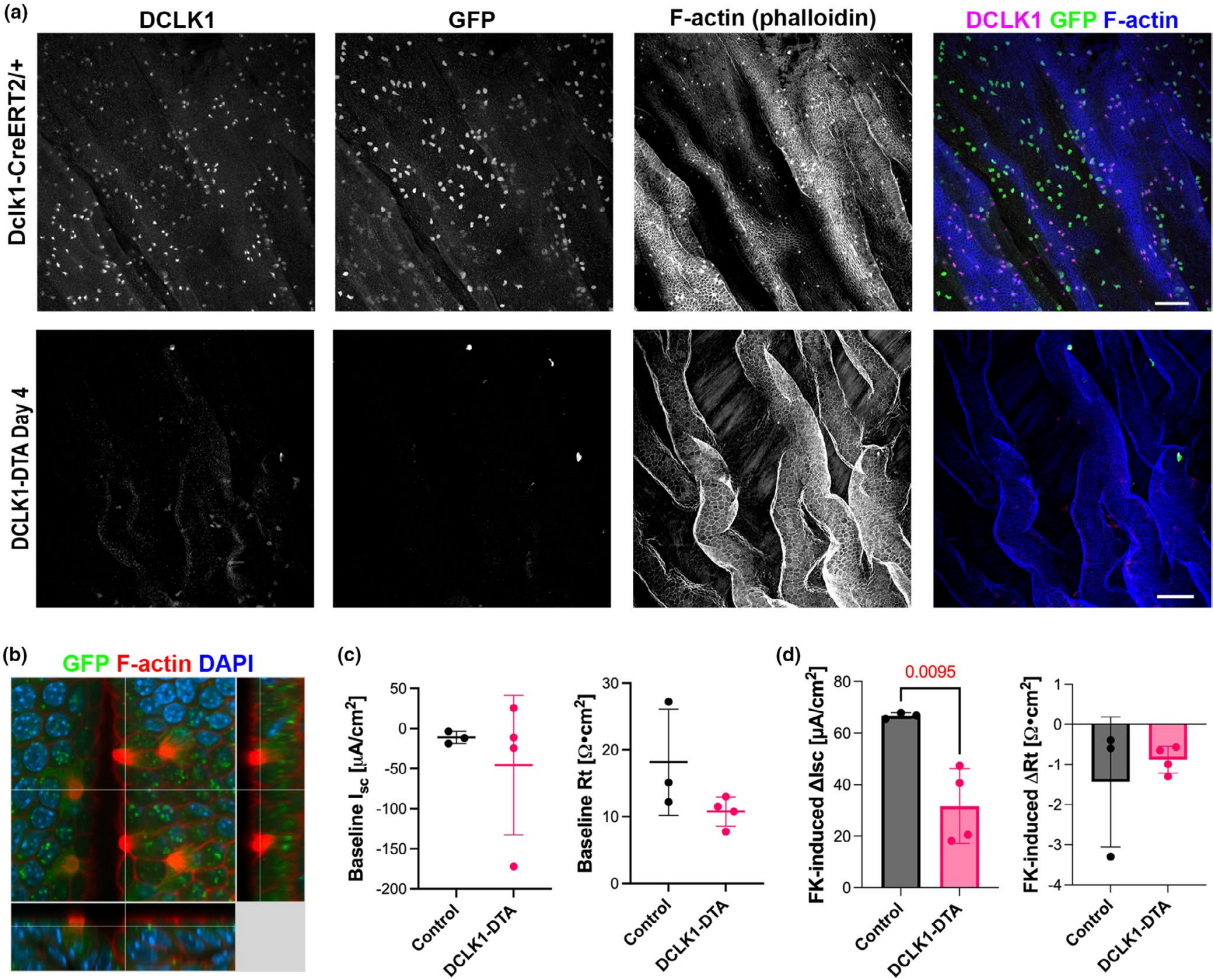
Acute depletion of biliary tuft cells in DCLK1‐DTA mice at Day 4. (a) Immunostaining for DCLK1 and GFP in whole‐mounted gallbladders 4 days post tamoxifen injection. F‐Actin and nuclei are stained with phalloidin and Hoechst, respectively. Scale bars = 50 μm. (b) Three‐dimensional reconstruction of confocal images of tuft cells in control mouse gallbladder. Dense F‐Actin microvilli and Actin rootlet structure are identified in GFP^+^ tuft cells. (c, d) Ion transport activities of gallbladders assessed by Ussing chambers. (c) Baseline *I*_sc_ and *R*_t_ had no significant difference. (d) Forskolin (FK) stimulates *I*_sc_ increase in control gallbladder larger than DCLK1‐DTA tissues (unpaired *t*‐test).

Gallbladder epithelium harbors bile acid‐sensing tuft cells, which have distinct transcriptional signatures from intestinal tuft cells and may influence digestive function (O'Leary et al., 2022). To assess whether biliary tuft cells are also affected in our DCLK1‐DTA mice, whole mount gallbladders were immunostained to identify tuft cells. Both DCLK1‐DTA mice and control littermates, which carry a heterozygous GFP knocked‐in allele (*Dclk1‐GFP‐CreER* ^*T2/+*^), were immunostained for GFP and DCLK1 (Figure 8a,b). Additional staining with phalloidin (F‐actin) identified dense “tuft” of microvilli and actin rootlet structures of biliary tuft cells similar to those of intestinal tuft cells (Burman & Kaji, 2021). *Dclk1*‐*GFP*‐*CreER* ^*T2/+*^ control tissues demonstrated GFP^+^/DCLK1^+^ tuft cells in the gallbladder and intestines, while DCLK1‐DTA mouse gallbladders harvested 4 days post‐tamoxifen treatment showed few tuft cells (Figure 8a). These observations indicate that this mouse model effectively diminishes tuft cell populations in both the biliary and intestinal epithelia. Steady state *I*_sc_ and *R*_t_ were found to be at similar levels in Ussing chambered murine gallbladders from control and DCLK1‐DTA mice (Figure 8c). Forskolin administration increased *I*_sc_ accompanied by a decrease in *R*_t_, likely representing chloride secretion (Figure 8d). This response was significantly reduced by tuft cell depletion, suggesting that biliary tuft cell loss may influence gallbladder function and affect nutrient absorption in the small intestine.

## 4 DISCUSSION

**FIGURE 9:**
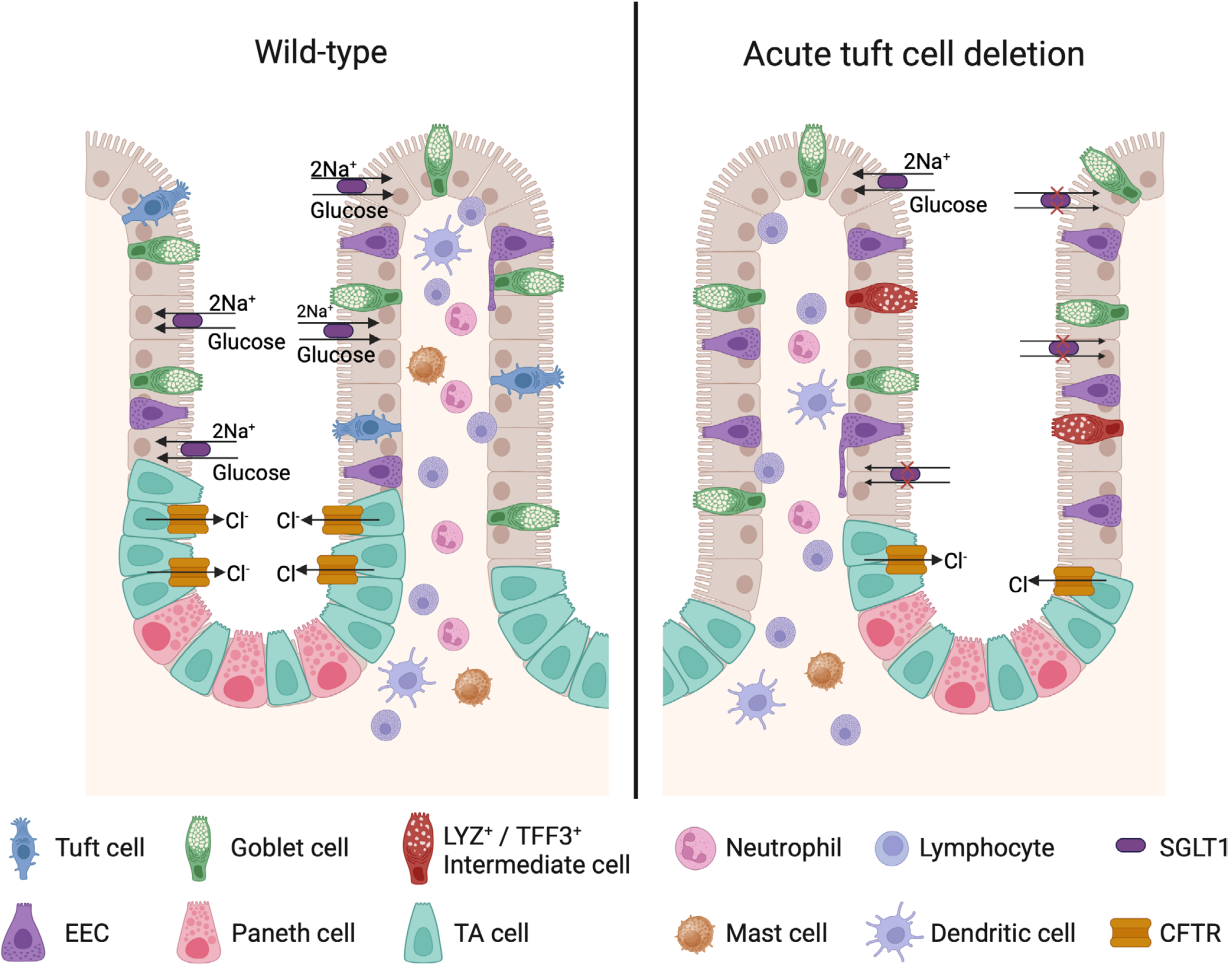
Summary of intestinal mucosal alterations induced by acute tuft cell deletion.

The tuft cell population is lost in several gastrointestinal diseases, and this loss is typically coincident with other deficits within the intestine, including malabsorption. In this study, we sought to highlight the effects of acute tuft cell deletion independent of a chronic disease state utilizing a Diphtheria Toxin subunit‐A (DTA) expression system in *Dclk1*‐expressing cells (DCLK1‐DTA). Following a single tamoxifen injection, intestinal and biliary tuft cell numbers were decreased in a time‐dependent manner, while DCLK1‐expressing enteric neurons were unaffected. Tuft cells were lost between 2 and 4 days after injection and recovered to normal levels by day 7. DCLK1‐DTA mice at Day 4 demonstrate significant changes in mucosal immune cell composition, an increase in aberrant secretory cell lineage differentiation, a decrease in crypt proliferation, and reduced sodium/glucose transporter activity along with small intestinal shortening (Figure 9). Our data suggest that acute tuft cell deletion correlates with body weight loss likely due to malabsorption within the murine small intestine.

Intestinal shortening is usually observed in colitis with additional hallmarks, including inflammation of the colon as well as overall body weight loss (Hu et al., 2021; Talley et al., 2021). In this study, however, we only observed a significant shortening in the length of the small intestine, with no difference in the length of the colon or any pathological signs of inflammation (Figures 1b–c and 7a–c). Additionally, both DCLK1^+^ enteric neurons and smooth muscle layers were unaffected by tamoxifen in DCLK1‐DTA mice on day 4 (Figure 1f). These observations suggest insufficient DTA expression under the *Dclk1* promoter in neurons and exclude a shortening of smooth muscles. Intestinal shortening following tuft cell deletion could explain the body weight loss observed due to reduced absorptive area as compared to controls (Figure 1a). The correlation of small intestinal length and tuft cell number has been identified in another mouse model: the deletion of A20, a deubiquitinase, which inhibits the release of IL‐13 from ILC2s, leads to constitutive activation of the type‐2 immune response and, subsequently, results in tuft cell hyperplasia (Garg et al., 2013). Consequently, in the A20‐deficient mice, tuft cell hyperplasia coincides with small intestine lengthening; the opposite of what was observed in our tuft cell deletion model. In the present study, exogenous recombinant mouse IL‐25 (mIL‐25) could overcome the phenotype of DTA‐induced tuft cell deletion in mice (Figure 1b). Supplementation with mIL‐25, which is secreted by tuft cells under homeostasis, to DCLK1‐DTA mice prevented the shortening of the intestine and body weight loss. This confirms that tuft cells play an extensive role in small intestinal remodeling, a relatively unexplored function of tuft cells. A significant decrease in proliferative cells in the small intestine after acute tuft cell deletion supports the hypothesis that tuft cells are important for maintaining intestinal epithelial proliferation. We hypothesize that compensation occurs in the germline tuft cell deletion (POU2F3^−/−^) model; however, further studies are required to identify the underlying mechanism (Figure 2a–f).

Nutrient absorptive function in the small intestine largely depends on the activity of transporters localized to the apical membrane of enterocytes. SGLT1 malfunction is implicated in the absorptive deficit of sodium, glucose / galactose, and water in the intestine (Martin et al., 1996). In our functional assays, DCLK1‐DTA mice demonstrated significantly impaired SGLT1 activity after acute tuft cell deletion without transcriptional changes in *Slc5a1* or *Tas1r2/Tas1r3* (Figure 3a–c,f–h). Currently, little is known about the connection between tuft cells and SGLT1 function or glucose metabolism. Tuft cells are positively correlated with improved glucose metabolism in obesity models (Chen et al., 2022). This function, as well as tuft cells’ involvement in maintaining the mucosal barrier function in the intestine (Nadjsombati et al., 2023), may impact SGLT1 activity on the apical membrane of enterocytes. The precise mechanism of SGLT1 regulation by tuft cells remains open for investigation. Portal hexose concentration was comparable between controls and DCLK1‐DTA mice, possibly due to a compensatory absorption of fructose, which is not mediated by SGLT1. Interestingly, portal lactate levels were significantly lowered by tuft cell loss, indicating a decreased energy precursor for the liver and systemic metabolism (Figure 3k). The loss of biliary tuft cells in the DCLK1‐DTA mice was observed in a similar time course to intestinal tuft cells and correlated to a decrease in the cAMP response (Figure 8). Biliary tuft cell defects are likely involved in a reduction of overall nutrient absorption and contributed to the body weight loss.

Active chloride excretion drives water secretion into the intestinal lumen (Keely & Barrett, 2022). This helps maintain intestinal barrier function and assists in parasite clearance (Billipp et al., 2024; Ndjim et al., 2024). Epithelial chloride secretion is mostly maintained by two intracellular signaling molecules, cAMP and Ca^2+^, and two membrane transporters, CFTR and CaCCs (Grubb, 1995; Guo et al., 2023). CFTR serves as the major source of chloride secretion in the murine intestine, and its disruption can lead to inflammation, dysbiosis, and cancer (Walker et al., 2022). Acutely deleting tuft cells led to reduced chloride secretion in the intestine; in particular, the CFTR‐dependent portion of our study (Figure 3e,f). This correlates to a reduction of crypt length in tuft cell‐deleted mice where CFTR is highly expressed (Figure 2a,b). Although there was no evidence of inflammation in our acute model, this observation may explain why the intestine is highly susceptible to inflammation upon tuft cell deletion, as previously reported with ChAT KO, Pou2f3 KO, and FLARE25 mice (Gerbe et al., 2016; Schutz et al., 2015; von Moltke et al., 2016). In CFTR KO mouse models, goblet cell hyperplasia in the intestinal mucosa is a secondary consequence of the absence of CFTR (Walker et al., 2022). In the present study, TFF3^+^ cells remained at relatively steady numbers between control and DCLK1‐DTA mice. Instead, we observed an increase in aberrant goblet/Paneth mixed cell lineage signatures in the small intestine (Figure 4a–f).

Goblet and Paneth cells are actively involved in protecting the intestinal barrier from invading pathogens. Goblet cell hyperplasia occurs during the type‐2 immune response to aid in the clearance of invading pathogens (Gerbe et al., 2016). In a LYZ‐1 deficient mouse model, the intestine maintains homeostasis by activating a type‐2 immune response, which is dependent on tuft cell expansion in the absence of Paneth cells (Yu et al., 2020). In our tuft cell depletion model, we observed an increase in lysozyme production in goblet‐like cells in villus regions (Figure 4b). Mature Paneth cells normally localize to the base of crypts, and mature goblet cells to upper villus regions, which do not typically express lysozyme. Stem cells differentiate into goblet and Paneth cells through Wnt/Notch signaling (Singh et al., 2022). Recent single‐cell RNA‐sequencing data have revealed that transcriptional differences between these two secretory cells are minimal, and there is likely a common precursor to Paneth and goblet cell lineages (Singh et al., 2022). The increased frequency of goblet/Paneth mixed‐type cells in the villi of DCLK1‐DTA mice at day 4 led us to suspect that these cells were immature secretory cells, in which terminal differentiation was disrupted by tuft cell loss. This phenomenon has also been seen in Sox‐9; Villin‐Cre mice, where tamoxifen‐induced conditional deletion of Sox9‐dependent Paneth cells in the intestine resulted in LYZ+ granules being detected in villus epithelial cells beyond their normal distribution in the crypts (Coutry et al., 2023). This staining pattern phenocopied in *Pou2f3−/−* germline KO mice, where we also observed these mislocalized LYZ^+^ cells in the villus (Figure 4c). This observation further bolstered our hypothesis that the rise in goblet/Paneth progenitor cells after tuft cell loss is likely a protective response. This is also supported by the increased frequency of enteroendocrine cells (EECs) in the DCLK1‐DTA mouse day 4 intestine (Figure 5a–d). EECs are another chemosensory cell lineage and immune response modulator (Yu et al., 2020). Cholecystokinin (CCK), a hormone secreted by a subset of EECs in response to luminal nutrients, is involved in resolving helminth infection (Worthington et al., 2013). CCK^+^ cell hyperplasia leads to reduced feeding, therefore reducing fat‐produced inflammatory adipokine leptin, which enhances type 2 immune responses to expel parasites from the small intestine. In the present study, specifically CCK^+^, but not 5‐HT^+^, GIP^+^, or SST^+^, EECs were significantly increased following tuft cell deletion (Figure 5c,d). This result highlights the potential for EECs to compensate for the void left by acute deletion of tuft cells.

Tuft cells trigger immune responses principally via IL‐25 secretion, which activates ILC2s in the lamina propria as a first step toward parasite eradication in the intestine (von Moltke et al., 2016). ILC2s, in addition to secreting IL‐13, also recruit macrophages, eosinophils, and granulocytes as part of the immune response to invading helminths (Artis & Spits, 2015; Saez et al., 2021). Under homeostasis, however, global tuft cell‐deficient (POU3F3−/−) mice demonstrate no differences in mucosal immune cell populations (Gerbe et al., 2016). In the present study, we observed a decrease in the number of mast cells and leukocytes in the intestine after acute tuft cell deletion, likely due to a loss of IL‐25 source (Figures 6 and 7). These observations suggest that tuft cells maintain some innate immune cell populations within the mucosa even when there is no parasitic presence in the intestine. Inevitably, this may also play a part in the increased susceptibility of the intestine, after tuft cell loss, to inflammation when challenged (Gerbe et al., 2016; Schutz et al., 2015; von Moltke et al., 2016).

In summary, this study shows that acute tuft cell deletion induces a host of small intestinal remodeling effects, highlighting new roles for tuft cells in nutrient absorption. We showed that tuft cell loss led to a decrease in body weight and a shortening of the small intestine in DCLK1‐DTA mice, which was restored by the administration of IL‐25. SGLT1 activity in the jejunum was hampered, highlighting a novel connection between tuft cells and SGLT1 function. Furthermore, reduced chloride secretion, decreased leukocytes, and mast cells showed an abridged ability for the intestine to respond to invading pathogens or allergen challenges. However, adaptive responses such as increased enteroendocrine cells, as well as the rise of an abnormal secretory lineage displaying goblet and Paneth cell markers, may compensate to restore intestinal homeostasis. Broadly, alterations in secretory cell lineages could serve as a focal point for understanding intestinal diseases and may provide insight into the treatment of such diseases.

## FUNDING INFORMATION

This work was supported by the National Institute of Health (NIH) grants R01 DK128190, RC2 DK118640, and Vanderbilt DDRC Pilot and Feasibility Program (P30 DK058404) to I.K. The DelGiorno laboratory is supported by the Vanderbilt Ingram Cancer Center Support Grant (P30 CA068485), the Vanderbilt‐Ingram Cancer Center SPORE in Gastrointestinal Cancer (P50 CA236733), the Vanderbilt DDRC (P30 DK058404), an American Gastroenterological Association Research Scholar Award (AGA2021‐13), NIH/NIGMS R35 GM142709, the Department of Defense (DOD W81XWH2211121), and Linda's Hope (Nashville, TN).

## CONFLICT OF INTEREST STATEMENT

The authors have no conflicts to disclose.

## ETHICS STATEMENT

The Institutional Animal Care and Use Committee (IACUC) of Vanderbilt University Medical Center, Nashville, TN, USA approved all experimental procedures and animal care (M2000104).
